# Infection remediation after septic device extractions: analysis of three treatment strategies including a 1-year follow-up

**DOI:** 10.3389/fcvm.2023.1342886

**Published:** 2024-01-11

**Authors:** Heiko Burger, Mona Strauß, Da-Un Chung, Manfred Richter, Tibor Ziegelhöffer, Samer Hakmi, Hermann Reichenspurner, Yeong-Hoon Choi, Simon Pecha

**Affiliations:** ^1^ Department of Cardiac Surgery, Kerckhoff-Klinik, Bad Nauheim, Germany; ^2^ Campus Kerckhoff-Klinik, Justus-Liebig-University Gießen, Bad Nauheim, Germany; ^3^ Department of Angiology and Cardiology, CardioVascular Center, Frankfurt/Main, Germany; ^4^ Department of Cardiology & Critical Care Medicine, Asklepios Klinik St. Georg, Hamburg, Germany; ^5^ Department of Cardiovascular Surgery, Asklepios Klinik St. Georg, Hamburg, Germany; ^6^ Department of Cardiovascular Surgery, University Heart and Vascular Center, Hamburg, Germany; ^7^ German Center for Cardiovascular Research (DZHK), Partner Site Hamburg/Kiel/Lübeck, Germany; ^8^ German Center for Cardiovascular Research (DZHK), Partner Site RhineMain, Frankfurt/Main, Germany

**Keywords:** outcome of septic lead extraction, treatment strategies, simultaneous CIED re-implantation, epicardial lead, pacemaker dependency, pocket infection, pacemaker endocarditis, follow-up

## Abstract

**Introduction:**

In CIED infections, all device material needs to be removed. But, especially in pacemaker-dependent patients it is often not possible to realize a device-free interval for infection remediation. In those patients, different treatment options are available, however the ideal solution needs still to be defined.

**Methods:**

This retrospective analysis includes 190 patients undergoing CIED extractions due to infection. Three different treatment algorithms were analyzed: Group 1 included 89 patients with system removal only (System removal group). In Group 2, 28 patients received an epicardial electrode during extraction procedure (Epicardial lead group) while 78 patients in group 3 (contralateral reimplantation group) received implantation of a new system contralaterally during extraction procedure. We analyzed peri- and postoperative data as well as 1-year outcomes of the three groups.

**Results:**

Patients in the system removal and epicardial lead groups were significantly older, had more comorbidities, and suffered more frequently from systemic infections than those in contralateral reimplantation group. Lead extraction procedures had comparable success rates: 95.5%, 96.4%, and 93.2% of complete lead removal in the System removal, Epicardial Lead, Contralateral re-implantation group respectively. Device reimplantation was performed in all patients in Epicardial lead and Contralateral reimplantation group, whereas only 49.4% in System removal group received device re-implantation. At 1-year follow-up, freedom from infection and absence of pocket irritation were comparable for all groups (94.7% Contralateral reimplantation group and Epicardial lead group, 100% System removal group). No procedure-related mortality was observed, whereas 1-year mortality was 3.4% in System removal group, 4.1% in Contralateral re-implantation group and 21.4% in Epicardial lead group (*p* < 0.001).

**Conclusion:**

In patients with CIED infection, systems should be removed completely and reimplanted after infection remediation. In pacemaker-dependent patients, simultaneous contralateral CIED re-implantation or epicardial lead placement may be performed, depending on route, severity and location of infection.

## 1 Introduction

The use of cardiac implantable electronic devices (CIED) has been an essential and indispensable therapy option for patients with symptomatic cardiac arrhythmias or a high risk of sudden cardiac death (SCD) for over 60 years. Unfortunately, for most patients, the initial implantation of such a device involves repeated revision procedures. Ideally, these revisions should be limited to only pulse generator replacement due to battery depletion. However, additional revision or correction surgeries do occur, stemming from technical problems with the implanted systems or infections.

The predominant local infections are pocket infections, which often occur shortly after device replacement, or those caused by percutaneous perforation of pulse generator or electrode components (1–3). In addition to local symptoms like pain, swelling, and tenderness, these infections pose a high risk for systemic seeding into the bloodstream. The transmission of the infection along the implanted electrodes is particularly feared, as it can lead to life-threatening endocarditis, fatal in 35% of cases if left untreated (4, 5). A similar vital threat can also arise from primary hematogenous bacteremia with superinfection of the implanted device components. Biofilm-forming bacteria, such as staphylococci, streptococci, or pseudomonas, are especially concerning in this context (6). This often leads to septic vegetations on the intravascular parts of the leads, triggering septic emboli and serving as a retreat for bacteria under a protective layer of biofilm during antibiotic therapy on the other. For these reasons, the timely and aggressive treatment of intracardiac infections is essential, as cardiac structures such as heart valves or myocardial tissue can be irreversibly destroyed if left untreated. Based on these findings, international and national professional societies recommend immediate and complete removal of intracardiac devices with a Class I recommendation in cases of proven infection (7–15).

However, a CIED cannot always be easily removed. In addition to technical and anatomical challenges, the further strategy must be carefully evaluated and planned, especially in pacemaker-dependent patients (16, 17). Additionally, there is still no uniform recommendation among experts regarding the timing of the re-implantation of a necessary system (12–19). Therefore, various approaches arise in clinical practice, which can be reduced to three established variants: The first and most commonly performed treatment option is system re-implantation after complete system removal with a time delay of 4–6 weeks under antibiotic therapy and after exclusion of an ongoing infection (18, 19). This strategy is limited by the requirement of an adequate intrinsic cardiac rhythm. In case of pacemaker dependency, as a second option, simultaneous implantation of an epicardial pacing lead through an additional left lateral thoracotomy (system removal and implantation of an epicardial pacing lead—EL) can be performed during removal of the infected system. This is then connected to a subcutaneously implanted “sacrificial pacemaker” and ensures continued stimulation (18). Alternatively, temporary percutaneous transvenous “sacrificial electrodes” can also be placed (18, 19). In both cases, implantation of a definitive system is performed in a second scheduled procedure after successful antibiotic therapy (18, 19). The third and final therapeutic option is a simultaneous implantation of a permanent system from the contralateral side after removal of the infected system (System removal and contralateral implantation of a new device—SI). Thus, uninterrupted full device therapy is possible—but with the remaining risk of re-infection, for example, via contact infections or continued hematogenous bacterial dissemination.

**Figure 1:**
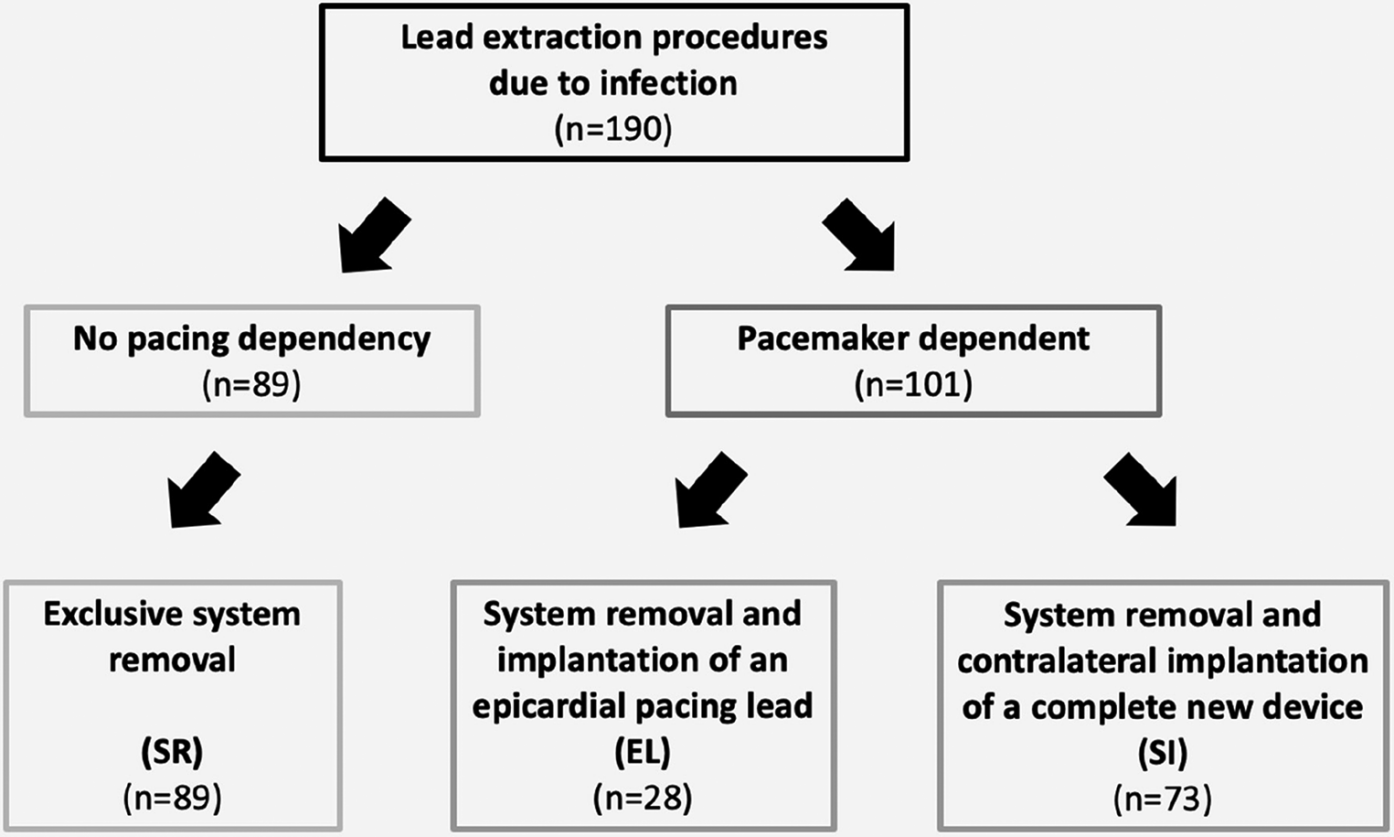
Selection of the study groups.

The decision for one of the described procedures is usually based on the clinical experience of the treating physicians, as there are no comparative studies or follow-up data on the outcome of these procedures in the currently available literature (13, 19). For this reason, we retrospectively analyzed all patients treated in our hospital between 2013 and 2019 who received a CIED removal/extraction due to device infection (Figure 1). We searched for differences in the pre-existing conditions of the patient groups in a retrospective analysis of the treatment pathways in order to evaluate the clinical decisions made. We further analyzed whether peri- and postoperative differences in the treatment courses during the hospital stay could be shown. Finally, we investigated the 1-year follow-up data of the 3 treatment strategies, in order to compare possible outcome differences between the treatment pathways.

## 2 Methods

The presented observation study is a retrospective analysis of all lead extractions performed in the Department of Cardiac Surgery of the Kerckhoff-Klinik Bad Nauheim between 2013 and 2019. We identified 190 extraction procedures in patients with infectious indications, which were divided into the three treatment paths described above (Figure 1). This resulted in a total of 89 procedures in which the systems were removed exclusively (System Removal Group), 28 procedures in which the system was removed and an epicardial pacing lead was implanted simultaneously (Epicardial Lead Group), and 73 patients in whom a completely new and definitive device system was implanted on the contralateral side (Contralateral reimplantation Group). Lead removal was performed by either manual traction after placement of a lead locking stylet or with use of laser- or mechanical rotational sheaths, if necessary.

In addition to patient-specific data, cardiac pre-existing cardiac conditions or treatments and relevant co-morbidities were recorded. Furthermore, the timing of the initial diagnosis, admission to our hospital, pre-operative antibiotic therapy, timing of the surgical procedure, and length of the treatment period were of interest. In particular, we also considered pre-operative infection parameters, previously identified pathogens, and the age of the implanted CIED components. Additionally, the indications for CIEDs and their implantation positions were recorded. During surgery, besides the group-specific method, the extraction techniques, number of removed electrodes, existing vegetation, pericardial effusion sizes, tricuspid valve function, and wound closure methods were documented along with the duration of the procedure, fluoroscopy, and laser times. In the post-operative course, the duration of the intensive care unit (ICU)- and overall hospital stay, further course of infection parameters (laboratory chemistry, pathogen detection), echocardiographic findings, and the discharge destination (home or another hospital) were recorded.

If a second surgery was required for re-implantation of a CIED, perioperative parameters and lead measurements were registered. At 1-year follow-up we reassessed the completed healing of the CIED pockets, device function, laboratory parameters, current NYHA class, LV-EF, and lead-specific measurements. Fatal treatment courses were also recorded and distinguished between perioperative and post-discharge time points.

The collected data were obtained from the digital and analog patient records of our hospital and, in individual cases, were supplemented with additional information from treating colleagues. All data were digitalized and anonymized after the data collection was completed. Finally, the statistical analysis, tabular and graphical processing, and evaluation of the results were performed.

The investigations were carried out in accordance with the Declaration of Helsinki of the World Medical Association on the ethical principles for medical research involving human subjects and were approved by the Ethics Committee of the State Medical Association of Hessen/Germany (reference number: 2022-3185-evBO).

### 2.1 Statistical methods

All statistical analyses were performed with SPSS statistical software version 21.0 (IBM Corp, Somers, NY, USA). Continuous values are expressed as mean ± standard deviation or standard error of mean (SEM) as indicated and were compared with Student's *t*-test after confirmation of normal distribution. Otherwise, the Mann–Whitney-*U*-test was used. Categorical variables are displayed as frequency and percentages and were compared using the Chi-square-test or Fisher's exact test in small sample sizes or when one or more of the cells had an expected frequency of five or less. Multi-group comparisons were performed using ANOVA test with Bonferroni *post-hoc* correction. For intra-group comparisons, a paired *t*-test was used. A *p*-value of less than 0.05 was considered to indicate statistical significance.

## 3 Results

### 3.1 Preoperative comparison of patient characteristics

The analysis of the three groups revealed significantly younger patients in the System removal group. However, in terms of gender and body dimensions, an equal distribution was observed throughout the entire patient population. The analysis of preprocedural parameters did not reveal any significant group-specific differences, but a detailed examination of the specific group data indicated tendencies.

Patients in the Contralateral reimplantation group had the lowest New York Heart Association (NYHA) classification of 2.4 and American Society of Anesthesiologists (ASA) classification of 3.0, as well as the lowest proportion of patients with diabetes (23.2%). They also exhibited the lowest level of renal dysfunction [creatinine 1.3 mg/ dl; glomerular filtration rate (GFR) 78.8 ml/ min/1.7], had the lowest incidence of coronary heart disease (45.5%), and the least frequent percutaneous coronary interventions (PCI) (21.9%) prior to surgery.

In contrast, patients in the Epicardial lead group had a higher NYHA class (2.5), the highest ASA class (3.4), and the lowest GFR (62.1 ml/ min/1.7) compared to the other groups. The highest percentage of arterial hypertension (82.1%) was also found in this group, along with the highest incidence of coronary heart disease (64.3%), which was reflected in the highest number of PCIs (39.3%) and cardiac surgeries (42.9%) prior to lead extraction.

The System removal group showed the highest NYHA class (2.6) and the second-highest ASA class (3.3), the highest creatinine level (1.6 mg/ dl), the second-worst GFR value (66.6 ml/ min/1.7), and the highest percentage of diabetics (34.8%). Unexpectedly, this group had the highest left ventricular ejection fraction (LV-EF) of all groups at 44.7% (Table 1).

**Table 1:** Patients’ baseline characteristics (*n* = 190).

	Exclusive system removal (SR)	System removal and implantation of an epicardial pacing lead (EL)	System removal and contralateral implantation of a new device (SI)	*P*-value
Patients [*n*]	89	28	73	
Mean age, years (range)	67.6 (31–93)	**72.1** **(38–86)**	70.4 (39–93)	**0**.**001**
Masculine sex, *n* (%)	64 (71.9)	**22** (**78.6)**	55 (75.3)	0.624
Mean BMI, kg/m2 ± SEM	27.3 ± 0.5	**27.5 ± 1.0**	27.0 ± 0.6	0.545
Clinical data				
NYHA classification, class (1–4) ± SEM	**2.6 ± 0.1**	2.5 ± 0.2	2,4 ± 0.8	0.332
ASA classification, class (1–5) ± SEM	3.3 ± 0.1	**3,4 ± 0.1**	3 ± 0.0	0.098
Left ventricular ejection fraction, % ± SEM	**44.7 ± 1.7**	39.4 ± 2.8	43.2 ± 1.6	0.126
Arterial hypertension, *n* (%)	64 (71.9)	**23** (**82.1)**	52 (71.2)	0.330
Diabetes mellitus, *n* (%)	**31** (**34.8)**	9 (32.1)	17 (23.3)	0.122
Creatinine, mg/dl ± SEM	1.6 ± 0.2	1,5 ± 0.1	1,3 ± 0.2	0.150
GFR, ml/min ± SEM	66.6 ± 3.8	62,1 ± 6.4	**78.8 ± 4.6**	0.058
Coronary artery disease, *n* (%)	44 (49.4)	**18** (**64.3)**	33 (45.2)	0.076
Previous PCI, *n* (%)	23 (25.8)	**11** (**39.3)**	16 (21.9)	0.086
Previous cardiac surgery, *n* (%)	19 (21.3)	**12** (**42.9)**	18 (24.7)	0.091
CABG, *n* (%)	8 (9.0)	**5** (**17.9)**	9 (12.3)	0.608
Heart valve surgery, *n* (%)	8 (9.0)	**5** (**17.9)**	6 (8.2)	0.173
CABG and valve surgery, *n* (%)	3 (3.4)	**2** (**7.1)**	3 (4.1)	0.592
Rhythm disease				
AV-block, *n* (%)	11 (28.2)	**9** (**69.2)**	24 (53.3)	**0**.**015**
Sick-sinus syndrom, *n* (%)	**28** (**71.8)**	4 (30.8)	21 (46.7)	0.092
ICD for primary prevention, *n* (%)	35 (70.0)	**11** (**73.3)**	20 (71.4)	0.564
ICD for secundary prevention, *n* (%)	**15** (**30.0)**	4 (26.7)	8 (28.6)	0.342
Severe heart failure (CRT indikation), *n* (%)	25 (28.1)	**15** (**54.0)**	24 (33.0)	**0**.**022**
Device informations				
Device type				
Pacemaker, *n* (%)	39 (43.8)	13 (46.4)	**45** (**61.6)**	**0**.**028**
ICD, *n* (%)	**50** (**56.2)**	15 (53.6)	28 (38.4)	**0**.**028**
CRT device among all CIED, *n* (%)	25 (28.1)	**15** (**53.6)**	24 (32.9)	0.070
System age				
Dwelling time pacemaker leads, years ± SEM	**8.6 ± 1.3**	6.4 ± 1.5	3.7 ± 0.6	**<0**.**001**
Dwelling time pacemaker leads >1 year, *n* (%)	**34** (**87.2)**	10 (76.9)	39 (86.7)	0.396
Dwelling time ICD leads, years ± SEM	6.1 ± 0.7	**8.7 **±** 1.4**	2.5 ± 0.3	**<0**.**001**
Dwelling time ICD leads >1 year, *n* (%)	42 (84.0)	**14** (**93.3)**	22 (78.6)	0.234
Device position				
Chest left, *n* (%)	53 (59.6)	14 (50.0)	41 (56.2)	0.861
Subpectoral, *n* (%)	34 (38.2)	8 (28.6)	26 (35.6)	0.644
Preoperative infection parameters				
Body temperature, °C ± SEM	36.3 ± 0.1	36.7 ± 0.2	36.0 ± 0.1	0.444
Blood cultures taken, *n* (%)	64 (71.9)	**26** (**92.9)**	26 (35.6)	**<0**.**0001**
Successful germ detection, *n* (%)	45 (50.6)	**22** (**78.6)**	18 (24.7)	**<0**.**0001**
Preoperative infection parameters of blood analysis				
Leukocytes, Ts/μl ± SEM	8.5 ± 0.3	11.2 ± 1.2	8.0 ± 0.4	**<0**.**0001**
CRP, mg/dl ± SEM	5.9 ± 0.9	6.3 ± 1.5	1.4 ± 0.3	**<0**.**0001**
PCT, mg/dl ± SEM	2.77 ± 1.3	3.19 ± 2.4	0.18 ± 0.1	**<0**.**0001**
Preoperative ultrasound results				
Left ventricular ejection fraction, % ± SEM	**44.7 **±** 1.7**	39.4 ± 2.8	43.2 ± 1.6	0.126
Intracardiac vegetations, *n* (%)	44 (49.4)	17 (60.7)	6 (8.2)	**<0**.**0001**
Pericardial effusion, mm ± SEM	1.6 ± 0.3	0.6 ± 0.3	0.8 ± 0.2	**<0**.**0001**
Tricuspid regurgitation, (degree 1–3) ± SEM	1.1 ± 0.1	1.2 ± 0.2	0.9 ± 0.2	0.983
Identified germs				
Gram positive coagulase positive coccus species, *n* (%)	30 (66.7)	9 (40.9)	9 (50.0)	
Gram positive coagulase negative coccus species, *n* (%)	12 (26.7)	9 (40.9)	7 (38.9)	
Gram positive lactobacillales, *n* (%)	5 (11.1)	4 (18.2)	2 (11.1)	
Gram negative proteobacteria, *n* (%)	1 (2.2)	0 (0.0)	2 (11.1)	
Gram positive enterobacteriaceae, *n* (%)	1 (2.2)	0 (0.0)	0 (0.0)	
Candida fungal diseases, *n* (%)	0 (0.0)	1 (4.5)	0 (0.0)	
Multiple infections, *n* (%)	4 (8.9)	1 (4.5)	2 (11.1)	
Route of infection				
Pocket infection and pocket perforation, *n* (%)	27 (30.3)	3 (10.7)	**54** (**74.0)**	**<0**.**0001**
Descending pocket infection, *n* (%)	25 (28.1)	**8** (**28.6)**	13 (17.8)	0.276
Bloodstream infection of various causes, *n* (%)	37 (41.6)	**17** (**60.7)**	6 (8.2)	**<0**.**0001**

Values are expressed as mean ± SEM or counts (*n*) and percentages (%). A *P*-value of <0.05 was considered statistically significant (bold written).

AV-Block, atrioventricular block; BMI, body mass index; CABG, coronary artery bypass graft surgery; CIED, cardiovascular implantable electronic device; CRP, C-reactive protein; GFR, glomerular filtration rate; CRT, implantable cardiac resynchronization therapy; ICD, implantable cardioverterdefibrillator; NYHA, New York Heart Association; PCI, percutaneous coronary intervention; PCT, procalcitonin; SEM, standard error of the mean.

### 3.2 Group comparison of preoperative rhythm disorders and device data

Comparing the underlying rhythm disorders, it could be observed that in the Epicardial lead group, there was a significantly higher proportion of atrio-ventricular (AV) block (69.2%), the highest proportion of primary prophylactic implantable cardioverter-defibrillator (ICD) patients (73.3%), and the highest proportion of cardiac resynchronization therapy (CRT) systems (54%). As expected, AV block was least common in the System removal group with 28.2%. Conversely, the proportion of sinus node disorders as an indication for pacemaker implantation was highest in this group. Furthermore, considering the type of implanted devices, it can be seen that in the System removal and Epicardial lead group, there was a comparable distribution between the implanted pacemaker (43.8% vs. 46.4%) and defibrillator systems (56.2% vs. 53.6%), while in the contralateral reimplantation group there was a significantly higher proportion of implanted pacemakers (61.6%).

When looking at the age of the implanted leads, we found the oldest pacemaker electrodes (8.6 years) in the System removal group, and the oldest defibrillator leads in the Epicardial lead group (8.7 years). In contrast, the ICD- and pacemaker leads with the shortest implant duration were seen in the contralateral reimplantation group (Table 1).

### 3.3 Preoperative infection analysis

Of particular interest in the patient analysis was the preoperative infection status. While none of the patient cohorts showed an elevated body temperature under initiated antibiotic therapy, statistically significant differences were detected in the frequency of collected blood cultures and positive pathogen detections. In advance, the most common blood samples (92.9%) were taken in the Epicardial lead group. In the System removal cohort, this measure was carried out in 71.9%, while it was only performed in the Contralateral reimplantation group in 35.6% of cases. Pathogens were most commonly detected in the Epicardial lead group (78.2%). Consistently, gram-positive, coagulase-positive cocci (66.7%/40.9%/50%) were predominantly found in all groups (System removal/Epicardial lead/Contralateral reimplantation), followed by gram-positive, coagulase-negative subspecies (26.7%/40.9%/38.9%). Gram-positive lactobacilli (11.1%/18.2%/11.1%) and gram-negative proteobacteria (2.2%/0%/11.1%) were also frequently detected in all groups, with slightly higher frequencies in the Contralateral reimplantation group than in the other groups. Multiple pathogens were seen in all groups, with the highest frequency (11.1%) in the Contralateral reimplantation group. Finally, blood analyses showed the highest inflammatory parameters in the Epicardial lead cohort (leukocytes: 11.2 10^3^/ µl, CRP: 6.3 mg/ dl, PCT: 3.2 ng/ dl), while the Contralateral reimplantation group showed the lowest signs of inflammation. Echocardiography was able to detect intracardiac lead vegetations most frequently in the Epicardial lead group (60.7%).

Furthermore, different primary sources of infection were identified in the groups. Isolated pocket infections were significantly more frequent in patients of the Contralateral reimplantation group (74%), while bloodstream infections represented the dominant etiology in the other two groups (Epicardial lead: 60.7%, System removal: 41.6%) (Table 1).

### 3.4 Peri- and post-operative findings

Perioperative data showed the highest proportion of patients requiring stimulation (75%) in the Epicardial lead group, with a high proportion in the Contralateral reimplantation group, while no patient required stimulation in the System removal group. Operating times varied depending on the surgical complexity, with the shortest operation times in the System removal group. Extraction procedures in all groups relied on the use of specialized extraction devices such as the excimer laser (46.6%–67.9%) or mechanical rotational extraction sheaths (7.1%–12.3%) in more than 50% of cases. On average, between 2.3 and 2.6 electrodes were removed per patient, with 93.2%–96.4% complete success rate. Existing lead vegetations were removed with an efficacy of 94.1% (Epicardial lead group) to 100% (Contralateral reimplantation group). Approximately one-quarter of System removal and Epicardial lead patients received a wearable cardioverter defibrillator (WCD) for bridging until ICD re-implantation. A second operation to *de novo* implant or complete an epicardial pacing system was performed in 49.4% (System removal group) and 39.3% (Epicardial lead group) of cases. Here, transvenous leads were added in 100% of cases, and in the EL group, 90.9% of epicardial leads implanted at index procedure could be re-used.

The necessary second implant procedure was performed in the System removal group at a median of 26 days after extraction, significantly earlier than in the Epicardial lead group (62 days). Most commonly, pacemaker and CRT-D systems were then implanted. Interestingly, 50.6% of System removal group patients did not receive a new device since there was no further indication for pacemaker/ICD device.

Surgical wounds could be primarily closed in 94.2% of all groups. Vacuum-assisted wound closure (VAC therapy) with the aim of secondary wound closure was used only in individual cases with the highest percentage in the System removal group (7.9%). Overall, there was only one case of a perioperative complication where myocardial rupture with hemorrhage occurred during implantation of an epicardial LV electrode. However, the complication was successfully treated and had no further long-term consequences (Table 2).

**Table 2:** Perioperative findings and postoperative course (*n* = 190).

	Exclusive system removal (SR)	System removal and implantation of an epicardial pacing lead (EL)	System removal and contralateral implantation of a new device (SI)	*P*-value
Patients [*n*]	89	28	73	
Perioperative heart rhythm				
Sinus rhythm, *n* (%)	78 (87.6)	3 (10.7)	28 (38.4)	**<0**.**0001**
Atrial fibrillation, *n* (%)	11 (12.4)	4 (14.3)	7 (9.6)	0.544
Pacemaker dependency, *n* (%)	0 (0.0)	21 (75.0)	38 (52.1)	**<0**.**0001**
Extraction technique used and results				
Simple traction, *n* (%)	34 (38.2)	7 (25.0)	30 (41.1)	0.168
Laser extraction, *n* (%)	46 (51.7)	19 (67.9)	34 (46.6)	0.075
Extraction by trepanation tools, *n* (%)	9 (10.1)	2 (7.1)	9 (12.3)	0.723
Intervention time, min ± SEM	59.4 ± 3.6	122.1 ± 9.1	127.0 ± 6.1	**<0**.**0001**
x-ray time, min ± SEM	3.1 ± 0.5	3.0 ± 0.5	10.4 ± 1.1	**<0**.**0001**
Laser pulses delivered, *n* ± SEM	4,251.5 ± 1,280	4,145.0 ± 932.2	5,266.9 ± 956.3	0.430
Number of leads removed, *n* ± SEM	2.3 ± 0.1	2.6 ± 0.2	2.6 ± 0.1	**0**.**019**
Leads completely removed, *n* (%)	85 (95.5)	27 (96.4)	68 (93.2)	0.989
Lead vegetations completely removed, *n* (%)	43.0 (97.7)	16.0 (94.1)	6.0 (100)	0.788
Primary wound closure, *n* (%)	82 (92.1)	27 (96.4)	70 (95.9)	0.548
Vacuseal vacuum bandage and two-stage wound closure, *n* (%)	7 (7.9)	1 (3.6)	3 (4.1)	0.514
Procedural complications, *n* (%)	0 (0.0)	1 (3.6)	0 (0.0)	0.246
New device implantation				
Simultaneous device implantation, *n* (%)	0 (0.0)	17 (60.7)	73 (100.0)	**<0**.**0001**
Second procedure to complete or re-implant a device, *n* (%)	44 (49.4)	11 (39.3)	0 (0.0)	**<0**.**0001**
No device reimplantation, *n* (%)	45 (50.6)	0 (0.0)	0 (0.0)	**<0**.**0001**
Epicardial lead parameter				
Left ventricular sense, mV ± SEM		10.9 ± 1.0		
Left ventricular impedance, Ohm ± SEM		527.8 ± 35.5		
Left ventricular pacing threshold, volt ± SEM		0.9 ± 0.1		
Bridging therapy				
Wearable cardioverter-defibrillator (WCD), *n* (%)	21 (23.6)	7 (25.0)		0.877
Second procedure to complete or re-implant a device, *n* (%)	44 (49.4)	11 (39.3)		0.391
Time between first and second intervention, median days [IQR]	26 [61.5]	62 [41.5]		**0**.**003**
Single chamber pacemaker, *n* (%)	1 (2.3)	0 (0.0)		0.930
Dual chamber pacemaker, *n* (%)	16 (36.4)	2 (18.2)		0.234
CRT pacemaker, *n* (%)	1 (2.3)	1 (9.1)		0.422
Single chamber ICD, *n* (%)	8 (18.2)	0 (0.0)		0.196
Dual chamber ICD, *n* (%)	3 (6.8)	0 (0.0)		0.663
CRT defibrillator, *n* (%)	15 (34.1)	8 (72.7)		0.300
Postoperative lead types				
Final use of transvenous leads, *n* (%)	44 (100.0)	11 (100.0)	73 (100.0)	
Further use of epicardial leads, *n* (%)		10 (90.9)		
Postoperative infection parameters of blood analysis				
Leukocytes, Ts/μl ± SEM	7.9 ± 0.3	9.1 ± 0.7	8.1 ± 0.3	0.068
CRP, mg/dl ± SEM	4.6 ± 0.6	4.9 ± 0.8	3.4 ± 0.5	0.201
PCT, mg/dl ± SEM	0.7 ± 0.4	0.5 ± 0.3	0.3 ± 0.2	0.194
Postoperative ultrasound results				
Intracardiac vegetations, *n* (%)	1 (1.1)	1 (3.6)	0.0 (0.0)	
Left ventricular ejection fraction (%) ± SEM	44.2 ± 1.7	41.2 ± 2.6	44.1 ± 1.6	0.453
Pericardial effusion, (mm) ± SEM	1.0 ± 0.3	0.4 ± 0.2	0.9 ± 0.2	0.985
Tricuspid regurgitation, (degree 1–3) ± SEM	1.1 ± 0.1	1.8 ± 0.5	1.3 ± 0.3	**<0**.**001**

Values are expressed as mean ± SEM, median [IQR] or counts (*n*) and percentages (%). A *P*-value of <0.05 was considered statistically significant (bold written).

CRP, C-reactive protein; CRT, implantable cardiac resynchronization therapy; ICD, implantable cardioverter-defibrillator; IRQ, interquartil range; mV, millivolts; PCT, procalcitonin; SEM, standard error of the mean; WCD, wearable cardioverter-defibrillator.

### 3.5 Procedure times and treatment endpoints required for therapy

The analysis of time intervals for diagnosis, initiation of therapy, hospital transfer, operative care, and the post-operative treatment period revealed that a significant amount of time had elapsed until patients received final surgical treatment in all groups. It took a median of 14 days (Epicardial lead group) to 19.5 days (System removal group) after diagnosis before patients were transferred to our hospital. Here, the process was expedited, and final surgical care could be provided after 1 day (System removal group) to 3 days (Epicardial lead group).

Postoperatively, none of the study groups had a prolonged intensive care unit stay (0–0.5 days), while the longest subsequent stay on the regular ward was seen in the Epicardial lead group with 14 days. Patients in the Contralateral reimplantation group were discharged home most frequently (84.9%), whereas only half of the other two groups were discharged home (System removal: 48.3%; Epicardial lead: 46.4%). All other patients had to be transferred to other hospitals for further treatment.

During the hospital stay, two patients (2.2%) in the Contralateral reimplantation group died from fulminant sepsis, which, in addition to terminal heart failure, developed into dialysis-dependent cardio-renal syndrome with right heart and liver failure and electrolyte imbalance. In the Epicardial lead group, three patients died (10.7%) during in-hospital stay. One patient died due to a fulminant pneumogenic septic event with dialysis-dependent anuria and multi-organ failure. Another patient developed a methicillin-resistant Staphylococcus aureus (MRSA) mediastinitis and an Enterococcus faecalis lead endoplastitis following a coronary artery bypass (CABG) and aortic valve operation. Despite the immediate removal of the foreign material, the septic process could not be averted, and the patient died in fulminant septic shock. A third end-stage heart failure patient with a Streptococcus sanguis pocket infection died in terminal heart failure following a primarily uncomplicated CRT system extraction due to the postoperative lack of biventricular pacing. In the Contralateral reimplantation group, there was only one death (1.4%). This occurred in a stimulation-dependent patient with renal failure who experienced an unclear gastrointestinal complication with severe vomiting following the primary uneventful removal of the system and contralateral device implantation. This resulted in cardiac arrest due to electromechanical uncoupling, leading to death (Table 3).

**Table 3:** Documented time intervals required for infection treatment (*n* = 190).

	Exclusive system removal (SR)	System removal and implantation of an epicardial pacing lead (EL)	System removal and contralateral implantation of a new device (SI)	*P*-value
Patients [*n*]	89	28	73	
Time intervals required for treatment				
Periode between infection diagnosis and antibiotic therapy, median days [IQR]	1.0 [7.5]	8.0 [16.5]	3.5 [12.3]	**<0** **.** **001**
Periode between diagnosis and transfer to LE-center, median days [IQR]	19.5 [27.8]	14.0 [24.0]	16.0 [59.0]	0.197
Periode between hospital admission to surgical treatment, median days [IQR]	1.0 [1.0]	3.0 [3.0]	2.0 [2.0]	**0**.**032**
In-hospital days				
Intensive care unit days, median [IQR]	0.0 [2.0]	0.5 [6.6]	0.0 [0.3]	**<0**.**001**
General ward days, median [IQR]	7.0 [7.3]	10.0 [13.0]	4.0 [4.0]	**<0**.**001**
Total number of postoperative in-hospital days, median [IQR]	8.0 [10.0]	14.0 [20.0]	5.0 [4.0]	**<0**.**001**
Total hospital days, median [IQR]	10.0 [10.0]	18.0 [20.0]	7.0 [5.0]	**<0**.**001**
Type of hospital discharge				
Discharge home, *n* (%)	43 (48.3)	13 (46.4)	62 (84.9)	**0**.**006**
Transfer to different hospital, *n* (%)	44 (49.4)	12 (42.9)	10 (13.7)	**0**.**012**
In-hospital mortality, *n* (%)	2 (2.2)	3 (10.7)	1 (1.4)	0.064
1-year follow up data (*n* = 84)				
Patients *n*, (% of the original collective)	46 (51.7)	19 (67.9)	38 (52.1)	
Pocket and device				
Wound healing without irritation, *n* (%)	46 (100.0)	18 (94.7)	36 (94.7)	0.877
Device pocket irritationless, *n* (%)	41 (100.0)	19 (100.0)	38 (100.0)	1.0
Proper generator function, *n* (%)	39 (95.1)	19 (100.0)	38 (100.0)	0.933
Follow-up infection parameters of blood analysis				
Leukocytes, Ts/μl ± SEM	7.0 ± 0.3	6.9 ± 0.3	7.2 ± 0.3	
CRP, mg/dl ± SEM	0.4 ± 0.1	0.5 ± 0.1	0.5 ± 0.2	
Lead parameter				
Atrial sense, mV ± SEM	3.7 ± 0.3	3.6 ± 0.5	2.7 ± 0.3	**0**.**004**
Atrial pacing threshold, V ± SEM	0.8 ± 0.1	0.6 ± 0.1	0.8 ± 0.1	0.454
Right ventricular sense, mV ± SEM	11.4 ± 0.7	11.0 ± 0.7	11.3 ± 0.4	0.988
Right ventricular pacing threshold, V ± SEM	0.9 ± 0.3	0.8 ± 0.1	0.7 ± 0.0	0.651
Left ventricular pacing threshold, V ± SEM	0.9 ± 0.1	1.2 ± 0.1	1.2 ± 0.2	0.348
Heart function				
NYHA classification, Class 1–4 ± SEM	1.7 ± 0.1	1.8 ± 0.1	1.8 ± 0.2	0.877
Left ventricular ejection fraction (LV-EF), % ± SEM	45.0 ± 2.2	44.3 ± 2.8	44.2 ± 2.5	0.664
Over all mortality				
Died during initial treatment, *n* (% related to the original collective)	2 (2.2)	3 (10.7)	1 (1.4)	0.064
Deceased during follow-up, *n* (% related to the original collective)	1 (1.1)	3 (10.7)	2 (2.7)	**0**.**042**
Total deceased in the entire observation period, *n* (% related to the original collective)	3 (3.4)	6 (21.4)	3 (4.1)	**0**.**006**

Values are expressed as mean ± SEM, median [IQR] or counts (*n*) and percentages (%). A *P*-value of <0.05 was considered statistically significant (bold written).

CRP, C-reactive protein; CRT, implantable cardiac resynchronization therapy; ICD, implantable cardioverter-defibrillator; IQR, interquartile range; LE, lead extraction; LV-EF, left ventricular ejection fraction; mV, millivolts; NYHA, New York Heart Association; PCT, procalcitonin; SEM, standard error of the mean; V, Volt; WCD, wearable cardioverter-defibrillator.

### 3.6 Patient outcome at 1-year follow-up

One-year follow-up was available in 103 out of the total of 190 treated patients (54.2%). The follow up was conducted as part of CIED interrogations, which amounted to 51.7% of the System removal group (*n* = 46), 67.9% of the Epicardial lead group (*n* = 19) and 51.2% of the Contralateral reimplantation group (*n* = 38). In this context, non-irritating wound conditions were found in 94.7% (Epicardial lead group, Contralateral reimplantation group) and 100% (System removal group), and generator pockets were irritation-free in 100% of all cases. In the three cases of irritating wound healing, the previous generator pocket with keloid formation or a superficial wound irritation was identified as source of discomfort. However, in no case further surgical measures were required. The new device implants demonstrated adequate device function in 100% of cases in the Epicardial lead and Contralateral reimplantation groups, whereas two uncomplicated RV electrode revisions were necessary in the System removal group due to loss of sensing (4.9%). Overall, all groups showed excellent lead parameter measurements after 1 year (Table 3).

Of particular interest was the final assessment of the treatment courses. The laboratory inflammatory parameters, LV-EF, and current NYHA class were again determined. It was found that the infection treatments in all groups were comparably effective and successfully completed. However, all patients showed a comparable improvement in NYHA classes and a recovered or improved LV-EF at the end of treatment. It is noteworthy that the LV-EF initially decreased in the two groups (Systemic removal group/Epicardial lead removal) without immediate implantation of a final system, while the heart function of the Contralateral reimplantation group continuously improved from the start of the intervention until the end of observation

Finally, the question arose regarding the number of lethal treatment courses. Using the social data, we were able to supplement the time interval between hospital discharge and the 1-year follow-up, although we could unfortunately only determine the date of death and not the exact circumstances of death. One death (1.1%) occurred in the 8th postoperative month in the System removal group, three (10.7%) occurred after one month and two months in the Epicardial lead group, and two (2.7%) occurred after one and 6 months in the Contralateral reimplantation group. The overall mortality rates at 1 year were 3.4% (System removal group), 21.4% (Epicardial lead group), and 4.1% (Contralateral reimplantation group), with the Epicardial lead group having the significantly highest mortality rate of all treated groups (Table 3).

## 4 Discussion

Device infections pose a significant clinical challenge, affecting a considerable proportion of patients. In Germany, they account for 10% (1,653 cases in 2020) of the 18,000 annual revision procedures, and globally, they constitute 1%–2% of interventions. International and national expert panels unanimously recommend the immediate and complete removal of infected systems (20, 21). However, there is a lack of universally accepted strategies for the timing of subsequent reimplantation. For instance, the EHRA “consensus document” acknowledges the absence of randomized trials on the appropriate timing of reimplantation.

Therefore, the timing and indication for reimplantation should be individually determined, with a reevaluation of the indication before the procedure. Reimplantation is advised no earlier than 72 h after retrieval, following the exclusion of persistent infection through blood culture-based testing. Baddour et al. suggests delaying reimplantation for proven valvular vegetations until at least 14 days after retrieval, with confirmation of negative blood cultures.

In cases where patients require continued pacing, recommendations include placing a contralateral percutaneous “sacrificial electrode” or implanting an epicardial electrode to minimize the risk of reinfection. However, these recommendations may have limited applicability in clinical practice, particularly for infected patients requiring ongoing pacing therapy or uninterrupted cardiac resynchronization therapy (CRT) for heart failure support.

In our hospital, treatment strategies were collaboratively determined by an interdisciplinary device team and an interdisciplinary endocarditis board. Decisions were based on the clinical assessment of symptoms, underlying arrhythmias, device dependencies, comorbidities, and the extent and location of infection foci. A retrospective analysis of these decisions revealed group-specific differences that tended to support the chosen treatment pathways.

For instance, the System removal group had no pacing-dependent patients but the highest number of implanted ICDs (56.2%), indicating the clinical significance of the infection event. The Epicardial lead group showed significant comorbidities in a severe infectious event, with the highest pacing dependence (75%) and the lowest left ventricular ejection fraction (LV-EF) (39.4%). This group also had the highest number of blood cultures (92.9%), microbe detection (78.6%), inflammatory parameters (leukocytes 11.2 10^3^/ µl, CRP 6.3 dl/ ml, PCT 3.2 ng/ dl), and intracardiac vegetations (60.7%), suggesting it was the most severely diseased cohort.

These observations suggested that this was the most severely affected group in our cohort, followed by the SR group. In our view, this justified, ex ante, our aggressive and invasive treatment strategies. In contrast, the Contralateral reimplantation group cohort appeared less severely affected, having the lowest NYHA (2.4) and ASA class (3.0), and showing fewer comorbidities (creatinine: 1.3 mg/ dl, diabetes mellitus: 23.3%, prior coronary artery disease: 45.2%, PCIs: 21.9%, cardiac surgery: 24.7%). Additionally, the lead dwelling time was significantly shorter (HSM: 3.7 years, ICD: 2.5 years), and in 74% of cases, the infection was limited to the generator pocket. These factors likely influenced the infrequent blood cultures and the few positive bacterial detections (24.7%). Thus, we concluded that this was the least severely affected study group with the best prognosis.

In our study, the infected material was removed in all groups with a class I/B indication according to the current expert recommendations (12–15). On average, 2.3–2.6 leads per patient were completely removed in 93.2%–96.4%. Interestingly, existing lead vegetations could be removed with the extraction instruments in 94.1%–100%, which may have had a positive effect on prognosis and treatment duration in our patient population.

Overall, there was only one periprocedural complication (Epicardial lead group), representing 0.5% of the total cohort. However, a total of 6 deaths (3.2%) occurred during hospitalization. Thus, there were fewer complications and deaths than expected based on the GALLERY registry (total complication: 4.3%; MAE: 2.1%; in-hospital mortality: 3.6%) or the ELECTRa study (total complication: 2.4%−4.1%; MAE: 1.7%) (22, 23). However, our study showed a slightly higher in-hospital mortality compared with the ELECTRa registry (ELECTRa: 1.2%−2.5%) (23). We attributed this mainly to significantly higher mortality in the Epicardial lead group and low case numbers (System removal group: *n* = 2%/2.2%; Epicardial lead group: *n* = 3%/10.7%; Contralateral reimplantation group: *n* = 1%/1.4%).

These findings raise the question of whether there are other, less invasive treatment options with a good prognosis for stimulation-dependent patients. One possibility is the insertion of a temporary transvenous “sacrificial electrode” or, alternatively, the implantation of a leadless pacemaker. Unfortunately, we could not include these options in our analysis because of the small number of cases. Nevertheless, it remains to be reported that the concept of the percutaneous “sacrificial pacemaker electrode” was initially criticized because of the risk of infection and dislocation (12, 18). However, publications reporting good results with this bridging method are now accumulating. Frausing et al. recently published the results of a nationwide Danish analysis on the incidence of infections after over 40,000 CIED implantations in which a temporary percutaneous pacing electrode was inserted for bridging. In the follow-up period of one year, there was no increased rate of all-cause CIED infections (24). Zhou et al. investigated the patient population of pacemaker-dependent CIED infections in the Temporary Pacing using Active Fixation Leads (TPAFL) study (25). In this study, a contralateral temporary stimulating electrode was implanted in 334 patients during the removal of an infected CIED system. Afterward, they received a new permanent system a median of 10 days later. There they observed a total of five adverse events (1.5%) and one infection (0.3%) in the entire cohort. Pecha et al. previously described comparable results in a smaller study in which there were even no reinfections or complications (26).

Regarding the implantation of leadless devices, most current publications refer to an approximately 30-day delayed LP implantation after the extraction of an infected CIED system—i.e., non-pacemaker-dependent patients—and report low reinfection rates (27, 28). In contrast, simultaneous implantation of an LP during an existing infection has been described only rarely and in small studies or individual case reports. For example, Chang et al. reported on 17 patients who received an LP for continued ventricular pacing during extraction of an infected device. Among these, no re-infection occurred after 143 days (29). Similar results were published by Tan et al. from a meta-analysis of patients receiving leadless pacemaker implantation after CIED infection. One hundred five patients were treated with concomitant LP-implantation with excellent outcomes and a low rate of device re-infection (0.4%) during mean follow-up of 11.3 months (30). Equal results were published by Mitachione et al. from an international LP registry, with low a LP-related major complication rate of 1.6% and mortality a rate of 5.4% (31). Equally hopeful results were provided by case reports such as Wu et al. (32) or Jacobs et al. (33), who found no re-infections after simultaneous implantation of an LP during extraction procedures.

Especially in younger pacemaker-dependent patients, leadless pacemaker implantation might provide an alternative treatment strategy. Good short- and mid-term results of LP implantation have been shown in younger patients in two previously published studies (34, 35). However, long-term outcomes need to be awaited and indications for leadless pacing need to be taken carefully into consideration.

Furthermore, a comparison of our findings with those of the prospective Multicenter Electrophysiologic Device Infection Cohort (MEDIC) study by Boyle et al. (36) in 434 patients seems to be of interest. Of these, 381 underwent extraction treatment, and 220 of them (57.7%) received new device systems after a median of 13 days. In comparison, significantly more patients (76.3%) received a new system in our overall cohort. Only in the Contralateral reimplantation group, the number was slightly lower (49.4%). However, Boyle's study did not focus on the outcome of different treatment strategies but rather on a possible correlation between the timing of device reimplantation after extraction and the frequency of re-infection. Six months after the initial extraction procedure, an overall re-infection rate of 11.3% and an overall mortality of 26.4% were observed, which our numbers could not confirm even after one year of follow-up.

Comparing similar groups in both studies, his cohort had 23 patients who, like our Contralateral reimplantation group (*n* = 73), received a new permanent CIED system during the extraction procedure. Six months later, 69.6% of his patients remained free from re-infection. Additionally, there was one re-infection (4.3%) and four patients (17.4%) died. In our study, however, we observed no re-infection after one year and three deaths (4.1%). Comparing our 1-year follow-up of the System removal group (*n* = 89, reimplantation 26 days after extraction) and our Epicardial lead group (*n* = 28, reimplantation 62 days after extraction) with Boyle's “reimplantation group” (*n* = 42, reimplantation 21 days after extraction), we would have expected a mortality rate of 14.3%, 2.4% of re-infections, and uncomplicated healing in 83.3%. In contrast, we found no re-infections in our study cohort. Additionally, our Systemic removal group performed significantly better with 100% uncomplicated wound healing and a mortality rate of 3.4% (*n* = 3). However, our Epicardial lead group had a high mortality rate of 21.4% (*n* = 6) after one year, which we attributed to the proven severe illness and the more invasive treatment with the epicardial lead and secondary system upgrade.

Overall, Boyle's group concluded that the risk of re-infection after complete removal of an infected system is very low regardless of the timing of reimplantation. We can confirm this statement with our retrospective data analysis. In addition, our long-term follow-up showed that irritation-free wound conditions were found in 94.7%–100%, and properly functioning CIEDs in 95.1%–100%.

**Figure 2:**
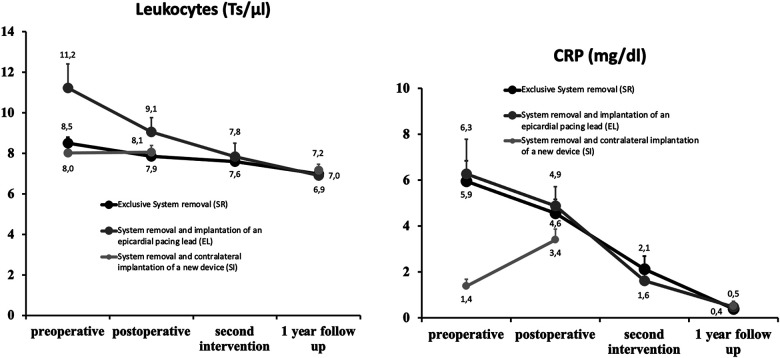
Left: Course of leukocyte level in the course of infection treatment. Right: Course of CRP level in the course of infection treatment. The mean values are shown with error bars as standard error of the mean (SEM).

**Figure 3:**
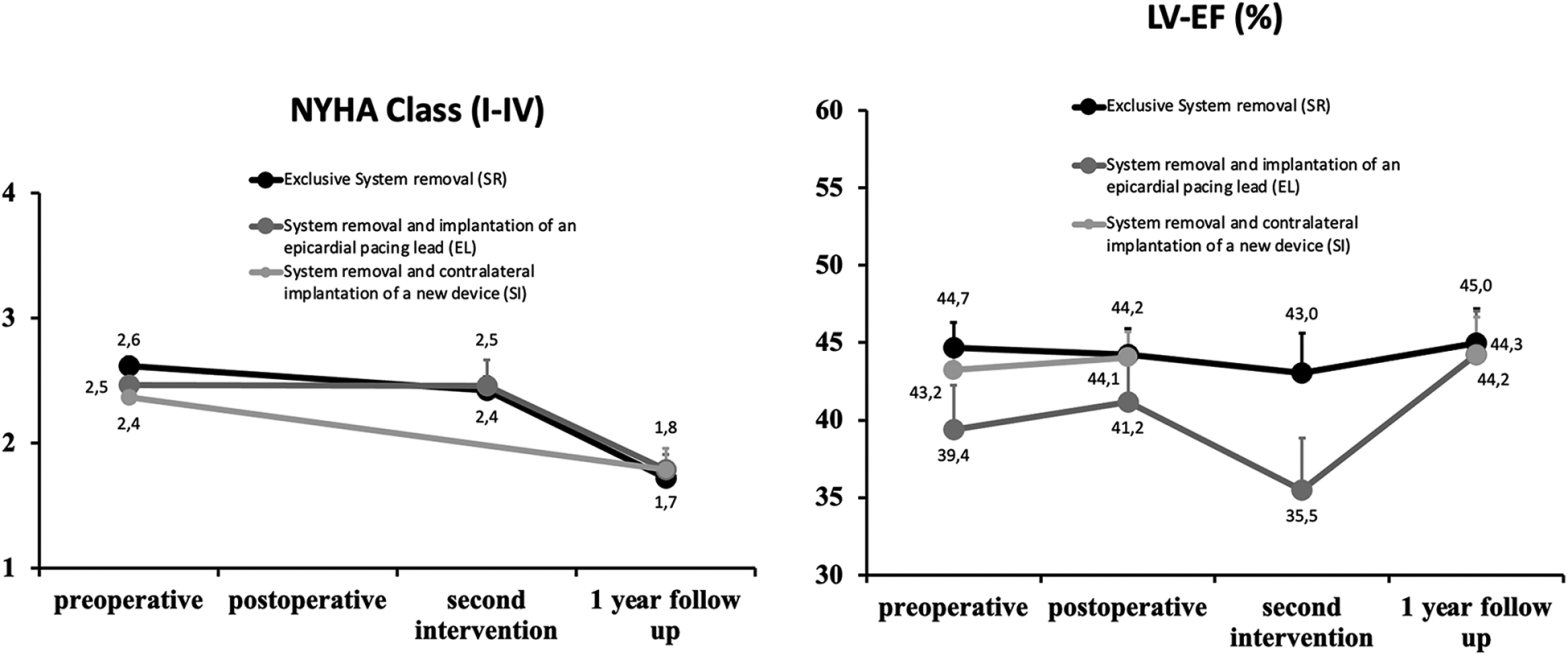
Left: Evolution of NYHA class during the course of infection treatment. Right: Development of LV-EF in the course of infection treatment. The mean values are shown with error bars as standard error of the mean (SEM).

Last but not least, the observations made in our study showed a significant decrease in inflammatory parameters (Figure 2) and improvement in NYHA classes as well as LV-EF (Figure 3) after one year in all groups, which we attribute to the healing of the infection. However, it was also shown that there was a transient decrease in LVEF in the Epicardial lead group due to the higher operative trauma of a lateral thoracotomy on the one hand, and in the System removal group due to the lack of adequate pacing on the other hand. However, LVEF increased again, even above baseline levels, after the implantation of a final system and resolution of the infection. These courses suggest that the clinical decisions made regarding method selection were appropriate.

However, significant delays in patient transfer from 14 to 19.5 days after diagnosis were also evident in our study. This delay could be due to difficulties in diagnosis, blood culture analysis, or organizational issues that cannot always be resolved quickly. On the other hand, the suspicion remains that a repeated attempt at purely conservative treatment was made, which is contrary to international recommendations (12, 36). Abandoning this approach could significantly improve outcomes. Upon arrival at the extraction center, the process accelerates, but still, there was a delay of 1–3 days. This was mostly due to poor-quality imaging or missing test results. To avoid these delays, we recommend that referring hospitals provide timely, up-to-date, high-quality test results—because removing infected devices within three days of diagnosis can significantly reduce in-hospital mortality rates (37, 38).

## 5 Limitations

The presented single-center study retrospectively analyzed patients from a clinical everyday population whose grouping was based solely on clinical assessment criteria. Thus, retrospective analysis looked for group-specific differences in collectives that were not fully comparable, which could result in a distorted picture. In addition, the fundamentally limited informative value of retrospective data analyses and observational studies should be pointed out, and last but not least, the small and unequal case numbers of the subgroups could cause a bias. Due to the limited number of patients multivariate analysis to determine outcome predictors was not possible. Use of Propensity score matching seems not to be reasonable in this patient collective since there are fundamental differences in the three groups.

## 6 Conclusion

The study authors were able to confirm that, in cases of severe bloodstream infections, complete removal of infected Cardiac Implantable Electronic Device (CIED) systems should be performed in accordance with international recommendations. In the absence of pacemaker dependency, our study demonstrated a favorable long-term prognosis with low mortality after two-stage reimplantation.

On the other hand, in pacemaker-dependent patients, treatment strategies should be carefully considered, taking into account infection routes and localization, implant age, and existing comorbidities. For instance, in cases of localized, non-systemic pocket infections, simultaneous implantation of a contralaterally implanted CIED system can lead to rapid recovery with a short hospital stay and low long-term mortality, yielding good outcomes. The authors found no significant differences in prognosis and reinfection rates between these two procedures in such cases.

In contrast, for patients with severe generalized bloodstream infections who are pacemaker-dependent, the implantation of an epicardial lead during extraction procedures proves to be a successful treatment option. However, mortality was significantly higher in this group during hospitalization and at the 1-year follow-up compared to other study groups—although it's essential to note that the patients in this group were also sicker. Whether the promising alternative of a temporarily implanted percutaneous pacing electrode or the implantation of a leadless pacemaker is a viable treatment option remains to be clarified in further studies.

## Data Availability

The original contributions presented in the study are included in the article/Supplementary Material, further inquiries can be directed to the corresponding author.
